# Characterization and Application of a Lytic Phage D10 against Multidrug-Resistant *Salmonella*

**DOI:** 10.3390/v13081626

**Published:** 2021-08-17

**Authors:** Zhiwei Li, Wanning Li, Wenjuan Ma, Yifeng Ding, Yu Zhang, Qile Yang, Jia Wang, Xiaohong Wang

**Affiliations:** 1 College of Food Science and Technology, Huazhong Agricultural University, Wuhan 430070, China; zhiweili@webmail.hzau.edu.cn (Z.L.); wanning.li@wondfo.com.cn (W.L.); wenjuanma@webmail.hzau.edu.cn (W.M.); yifengding@webmail.hzau.edu.cn (Y.D.); 123zy@webmail.hzau.edu.cn (Y.Z.); yangqile@webmail.hzau.edu.cn (Q.Y.); wangjia@mail.hzau.edu.cn (J.W.); 2 Key Laboratory of Environment Correlative Dietology, Huazhong Agricultural University, Wuhan 430070, China

**Keywords:** *Salmonella*, multidrug-resistant, phage, genomic, cocktail, lysis, eggs

## Abstract

*Salmonella* is a widely distributed foodborne pathogen that is a serious threat to human health. The accelerated development of drug resistance and the increased demand for natural foods invoke new biocontrol agents to limit contamination by multidrug-resistant (MDR) *Salmonella* strains. In this study, a lytic *Salmonella* phage named D10 was characterized at the biological and genomic levels. D10 possesses a short latent period (10 min) and a large burst size (163 PFU/cell), as well as adequate stability under a range of pH conditions and moderate thermal tolerance. D10 effectively lysed different MDR *Salmonella* serovars and repressed their dynamic growth in the medium. Genomic analysis disclosed that D10 is a new member of the *Siphoviridae* family and lacks the genes implicated in lysogeny, pathogenicity, or antibiotic resistance. A three-ingredient phage cocktail was then developed by mixing D10 with previously identified myovirus D1-2 and podovirus Pu20. The cocktail significantly reduced the count of MDR strains in liquid eggs, regardless of the temperature applied (4 and 25 °C). These results suggest that phage D10 is a promising tool to prevent food contamination by MDR *Salmonella*.

## 1. Introduction

*Salmonella* is a widely distributed foodborne pathogen that causes a variety of clinical symptoms, such as diarrhea and fever [1]. It has been estimated that *Salmonella* is associated with 1.35 million infections, 26,500 hospitalizations, and 420 deaths annually in the U.S. [2]. In the EU, 87,923 human salmonellosis cases were reported in 2019, with 926 salmonellosis foodborne outbreaks being observed. *Salmonella enterica* serovars Enteritidis (*S*. Enteritidis) and Typhimurium (*S*. Typhimurium) are the most commonly recovered [3].

Eggs and egg products accounted for around 37% of salmonellosis outbreaks in the EU in 2019 [3]. These foods have been listed by the U.S. Food and Drug Administration (FDA) as one of the riskiest agents in regard to *Salmonella* infections [4]. Due to the difficulty in removing *Salmonella* colonizers from animal hosts [5], effective strategies directly targeting risky foods are urgently needed.

In recent years, with the development of the modern food and livestock industry, the use of antibiotics has been increased, resulting in the spread of drug resistance [5]. In 2019, drug-resistant *Salmonella* was classified by the Centers for Disease Control and Prevention (CDC) as a serious threat to human health [6]. It has been revealed that multidrug-resistant (MDR) *Salmonella* strains are responsible for at least 100,000 *Salmonella* infections each year and are related to several outbreaks worldwide [5,7,8]. This emphasizes the necessity to develop antibiotic-free solutions for the control of MDR *Salmonella* strains. Despite the development and application of physical methods, such as irradiation, pasteurization, and high hydrostatic pressure in food processing, concerns have been raised, since some of these methods can cause undesirable changes in the nutritional and sensory qualities of food. Moreover, it is difficult to build a unit for high hydrostatic pressure [9].

Since being discovered in the last century, phages have been clinically applied to treat bacterial infections [10]. In the last decade, due to the rapid rise in MDR bacteria, phages have received mounting attention to replace or complement antibiotic therapy because they are specific, safe, and effective in the biocontrol of a variety of bacterial pathogens [10,11,12]. Additionally, phages have a negligible influence on food nutrients and flavors [13], rendering them a promising alternative to chemical additives in food preservation and processing. Despite the number of *Salmonella* phages being reported and tested, the current phage resource library is still limited in the context of the accelerated spread of drug resistance and the increased demand for natural foods [5,14].

Herein, we characterize a lytic *Salmonella* phage (D10) isolated from raw chicken by evaluating its biological properties, including its morphology, growth, and resistance to different temperature and pH conditions. The antimicrobial effect of phage D10 on different MDR *Salmonella* strains was tested in culture medium. Whole-genome sequencing of D10 was conducted to obtain more detailed information on its genome. Furthermore, D10 was used to develop a three-ingredient phage cocktail with two previously identified *Salmonella* phages, D1-2 (*Myoviridae*) and Pu20 (*Podoviridae*) [15,16]. The feasibility and effectiveness of the cocktail in the biocontrol of prevalent MDR *S*. Enteritidis and *S*. Typhimurium were measured in liquid eggs at 4 and 25 °C. These data suggest that phages and phage cocktails are promising agents for controlling MDR *Salmonella* strains, even in complex food matrices.

## 2. Materials and Methods

### 2.1. Salmonella Strains and Culture Conditions

Bacterial information is summarized in Table S1. *Salmonella enterica* serovar Dublin 3723 (*S*. Dublin 3723) was employed as the host strain to isolate phage D10. The *Salmonella* strains used for determining the lytic activity of D10 were recovered from food or clinical samples and were measured to be resistant to multiple antibiotics. The drug resistance profiles of the two strains used for the biocontrol of *Salmonella* in liquid eggs (*S*. Enteritidis 11561 and *S*. Typhimurium SJTUF 13277) are described in Table S1. The strains used in this study were stored in glycerol at −80 °C and were grown in Lysogeny Broth (LB) at 37 °C as needed.

### 2.2. Isolation of Salmonella Phage D10

The *Salmonella* phages presented in this study were previously isolated and purified by our group from various sources [15]. Briefly, after filtering, samples were mixed with bacterial suspensions in 2×YT broth and were then cultured at 37 °C with shaking (160 r/min) for 12–18 h. The mixture was then subjected to centrifugation at 8000 r/min for 15 min (Allegra X-30R Centrifuge, Beckman Coulter, Shanghai, China), followed by filtrating the supernatant using 0.22 μm filters. Subsequently, the filtrate (10 μL) was inoculated onto double-layer agar plates (the bottom layer was LB with 1.5% agar and the overlay was LB with 0.7% agar containing host strain suspensions). After incubation at 37 °C, clear plaques on the plate were picked and cultured with the host strain (100 μL) in 1 mL of 2× YT broth at 37 °C (12–18 h). The culture was then centrifuged and the supernatant was filtered to obtain phage filtrates. The phages were purified using the double-layer agar plate method. Briefly, the serially diluted phage filtrates (100 μL) were mixed with host strain suspensions (100 μL) and molten 0.7% LB agar (3.5 mL). Then, the mixture was added onto the 1.5% LB agar plate and cultured overnight at 37 °C. Individual plaques were picked and cultured with the host strain in 2× YT broth at 37 °C for 12–18 h. Subsequently, the culture was centrifuged and the supernatant was filtered to obtain purified phages. The purification was repeated until the lytic plaques became homogeneous. The phages were preserved in glycerol at −80 °C. Examination of the host range of isolated phages was performed using a spot test through the double-layer agar plate, and the raw data were first published in our previous study [15]. Clustering analysis of the quantitative features of phage-induced plaques was performed using R package pheatmap version 1.0.12 [17].

### 2.3. Lytic Effects of D10 on MDR Salmonella

The lytic activity of D10 to the MDR strains recovered from the different samples was measured as previously described [18]. For this, the phage lysate (5 μL) was inoculated onto the double-layer agar plate containing target *Salmonella* isolates and was cultured overnight at 37 °C. The lytic activity was quantified by evaluating phage-induced plaques using a validated scoring method, where numbers 0, +1, +2, +3, and +4 represent no lytic zone, an opaque zone, a partially clear zone, a generally clear zone, and a completely clear zone, respectively [19].

### 2.4. Morphology and Structural Protein Analysis

Phages were centrifuged (40,000 r/min) for 1 h and were then suspended in 0.1 mol/L of ammonium acetate. The copper grid for transmission electron microscopy (TEM) was incubated with the phage suspension for 10 min and was then stained for 10 min using a phosphotungstic acid solution [15]. The phage was observed by TEM (Hitachi H-7000FA, Tokyo, Japan) and characterized by Digital Micrograph Demo 3.9.1. 

The D10 particles were extracted and enriched according to our previously established method [16]. The phage protein was analyzed by sodium dodecyl sulfate polyacrylamide gel electrophoresis (SDS-PAGE).

### 2.5. Adsorption and One-Step Growth Curve

The adsorption and one-step growth curve were measured as previously described with modifications [20]. For the adsorption, the lysate of D10 (5 mL) was added into the *S*. Dublin 3723 culture (5 mL) at an MOI of 0.01, followed by shaking incubation at 37 °C for 20 min. During this time, the suspension (300 μL) was collected in 5 min intervals and incubated on ice for 30 s, followed by centrifugation at 7000 r/min for 30 s. The phage titer in the supernatant was then calculated by the double-layer agar plate method. The adsorption rate was calculated as (initial phage titer—final phage titer)/initial phage titer.

For the one-step growth curve, the D10 lysate (500 μL) was added into the *S*. Dublin 3723 culture (500 μL) at an MOI of 0.01, followed by shaking incubation at 37 °C for 20 min. Subsequently, the mixed suspension was centrifuged at 7000 r/min for 2 min. Then, the pellet was washed two times and suspended with preheated LB (10 mL). The resuspended mixture was incubated at 37 °C for 3 h with shaking (160 r/min). During this period, 300 μL of the mixture was collected in 10 min intervals, with subsequent centrifugation at 7000 r/min for 30 s. The phage titer was calculated by the double-layer agar plate method. The burst size was determined by dividing the phage titer at the plateau phase by the initial count of *S*. Dublin 3723.

### 2.6. Thermal and pH Resistance

To evaluate the thermal tolerance of D10, 1 mL of 10^7^ PFU/mL of the phage suspension was incubated at different temperatures for 30 or 60 min. Similarly, for the pH stability, 100 μL of 10^8^ PFU/mL of D10 lysate was mixed with 900 μL of LB with a pH ranging from 2 to 13, followed by incubation at 37 °C for 2 h. Subsequently, for both parameters, the phage titer was measured by the double-layer agar plate method.

### 2.7. Inhibition of the Dynamic Growth of MDR Salmonella by D10

For the inhibitory activity of D10 to the dynamic growth of MDR strains, 100 μL of 10^5^ CFU/mL of *S*. Enteritidis 11561 or *S*. Typhimurium SJTUF 13277 was incubated with D10 lysate (100 μL) ranging from 10^3^ to 10^8^ PFU/mL at 37 °C for 12 h. The bacterial growth was monitored via determining the OD_600_ at 1 h intervals. LB mixed with the bacterial suspension was used as the positive control, whilst LB mixed with 10^7^ PFU/mL of D10 lysate was used as the negative control.

### 2.8. Genomic Features of D10

The concentration of D10 genomic DNA was measured using the Qubit fluorometer (Thermo Fisher, Waltham, MA, USA). Genome sequencing was conducted on the Illumina HiSeq platform (Illumina, San Diego, CA, USA) using 2 × 150 bp paired-end runs, followed by assembly through MicrobeTrakr plus 0.9.1 [21]. Phage genes were identified by Prodigal 2.6.0 and annotated using MyRast [22], followed by manual verification through BLASTP [23] and Uniprot [24]. The CGView Comparison Tool [25] and the Easyfig Tool [26] were employed to create circular and linear genome maps, respectively. Phylogenetic analysis based on the terminase large subunit was conducted using MEGA X with the maximum likelihood method and 500 bootstraps [27] and was depicted via FigTree (http://tree.bio.ed.ac.uk/software/figtree/ (accessed on 21 June 2020)). Potential virulence factors, antibiotic resistance genes, and tRNAs were detected by the Virulence Factor Database [28], Comprehensive Antibiotic Resistance Database [29], and tRNAScan-SE [30], respectively. Genome sequences of phages LPST10, VB_StyS_BS5, and KFS-SE2 are available in the National Center for Biotechnology Information (NCBI) database with accession numbers KY860935, MN692673, and MK112901, respectively.

### 2.9. Biocontrol of Salmonella in Liquid Eggs by the Three-Ingredient Phage Cocktail

The three-ingredient phage cocktail was prepared by mixing equal volumes of 1 × 10^9^ PFU/mL of lysates of myovirus D1-2 [15], podovirus Pu20 [16], and the currently identified siphovirus D10. Egg samples were cleaned with distilled water and 75% ethanol, followed by sterilization under UV light for 30 min. The liquid egg whites and egg yolks were homogenized using sterilized glass rods. The sterility was verified by spotting liquid eggs onto LA plates and incubating the plates at 37 °C. Then, the sterile liquid egg whites or liquid egg yolks (9.8 mL) were mixed with *S*. Enteritidis 11561 or *S*. Typhimurium SJTUF 13277 suspension (100 μL; 10^5^ CFU/mL) and incubated at 4 or 25 °C for 20 min. Subsequently, the D10 lysate (100 μL; 10^8^ or 10^9^ PFU/mL) was mixed into the mixture and incubated at 4 or 25 °C. The viable bacteria were quantified at 0, 1, 3, 6, 12, and 24 h after incubation by serial dilution [15].

### 2.10. Statistical Analysis

Statistical analysis was conducted using Prism 8.0 (GraphPad Software, La Jolla, CA, USA). The level of statistical significance was designated at *p* < 0.05 and was determined by the two-way ANOVA with Tukey’s multiple comparison.

## 3. Results

### 3.1. Salmonella Phage D10 Shows High Lytic Activity

**Figure 1:**
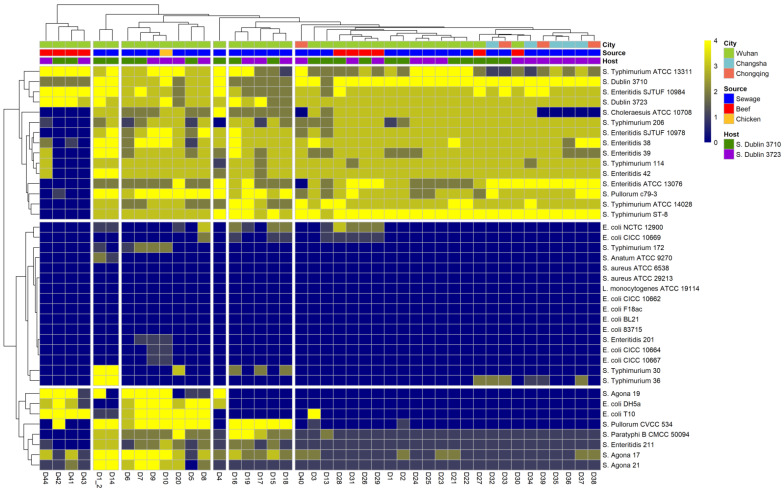
Clustering analysis of the quantitative features of phage-induced plaques. The lytic activity is presented by numbers and visualized by colors from yellow to blue: “+4” denotes a completely clear zone; “+3” denotes a generally clear zone with a faint hazy background; “+2” denotes obvious turbidity throughout a clear lytic zone; “+1” denotes an individually opaque zone; “0” denotes no lytic zone. The quantitative features of the plaques were hierarchically clustered using the Euclidean distance.

*Salmonella* phage D10 was isolated using the host strain *S*. Dublin 3723. Cluster analysis of the quantitative characteristics of the phage-induced plaques revealed that D10 was allocated to a highly lytic cluster (Figure 1). D10 lysed 23 out of 26 tested *Salmonella* strains from different serovars. Conversely, it was unable to infect bacteria from *Listeria monocytogenes* and *Staphylococcus aureus* (Figure 1). Moreover, D10 was the only phage isolated from raw chicken; therefore, D10 was chosen for further study.

### 3.2. Characterization of Phage D10

**Figure 2:**
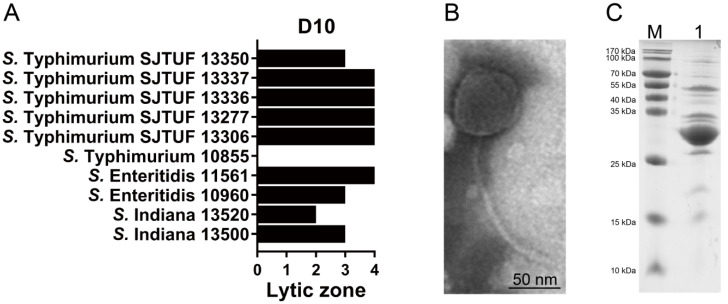
Characterization of phage D10. (**A**) The lytic activity of D10 to multidrug-resistant (MDR) *Salmonella* isolates with scoring on lytic zones: “+4” denotes a completely clear zone; “+3” denotes a generally clear zone with a faint hazy background; “+2” denotes obvious turbidity throughout the clear lytic zone; “+1” denotes an individually opaque zone; “0” denotes no lytic zone. (**B**) TEM-based morphology of D10 with a bar indicating a magnification size of 50 nm. (**C**) Phage proteins separated by SDS-PAGE. M: marker; lane 1: D10.

To confirm the ability of D10 to kill infection-associated bacteria, MDR *Salmonella* strains recovered from food or clinical samples were used for the antibacterial test, which indicated that D10 can infect and lyse 9 out of the 10 examined isolates (Figure 2A). TEM-based morphology of D10 showed a typical regular polyhedral head with a diameter of around 62 nm (Figure 2B). This also depicted a phage tail with a length of around 161 nm and a diameter of around 5 nm (Figure 2B), which suggests that D10 is a member of the *Siphoviridae* family. Phage particles were analyzed by SDS-PAGE to determine the structural proteins, where at least eight bands emerged, ranging from 27 to 100 kDa, with the most abundant band of around 30 kDa, presumably corresponding to the major capsid protein (Figure 2C).

### 3.3. Growth and Stability of D10

**Figure 3:**
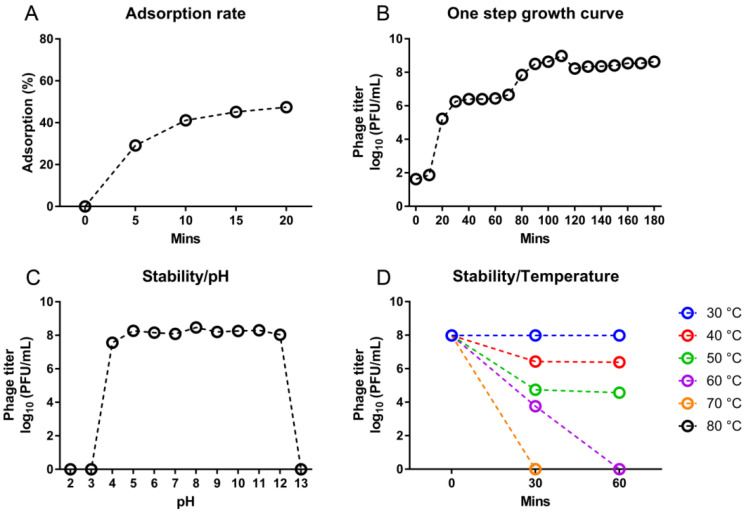
Growth and stability of phage D10. (**A**) Adsorption rate. (**B**) One-step growth curve. (**C**) Stability of D10 under different pH conditions. (**D**) Stability of D10 at temperatures ranging from 30 to 80 °C.

The adsorption rate of D10 was determined using *S*. Dublin 3723. At 20 min after incubation, an adsorption of 47.4% was detected (Figure 3A). The one-step growth curve of D10 presented a latent period of 10 min and an exponential growth stage from 10 to 110 min with a burst size of 163 PFU/cell (Figure 3B). D10 was able to be active when subjected to the condition of pH 4 to 12; however, it became completely inactive at pH 3 or 13 (Figure 3C). Different from high pH stability, D10 showed moderate thermal tolerance; it was partially and completely inactive at 40 and 70 °C for 30 min, respectively (Figure 3D).

### 3.4. Inhibition of Dynamic Growth of MDR Salmonella by D10

**Figure 4:**
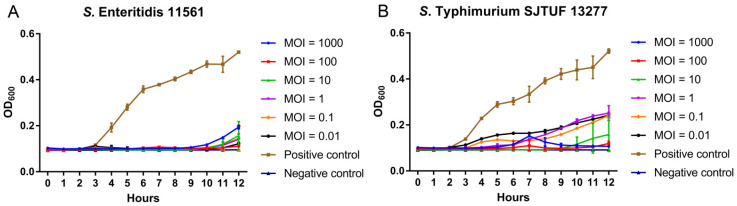
Inhibition of the dynamic growth of MDR *Salmonella* by phage D10 in broth medium. (**A**) Inhibitory effects of D10 on the growth of *S*. Enteritidis 11561 at MOIs ranging from 0.01 to 1000. (**B**) Inhibitory effects of D10 on the growth of *S*. Typhimurium SJTUF 13277 at MOIs ranging from 0.01 to 1000.

We then measured the inhibitory effect of D10 on the dynamic growth of *S*. Enteritidis 11561 and *S*. Typhimurium SJTUF 13277, two MDR strains isolated from food samples. In the medium, these *Salmonella* strains continuously grew from 3 to 12 h post-inoculation when lacking D10 treatment (Figure 4A,B). When D10 was added at MOIs ranging from 0.01 to 1000, it successfully diminished the growth of both MDR strains, despite the mild difference in the inhibitory pattern between the two strains (Figure 4A,B). For *S*. Enteritidis 11561, D10 completely inhibited the bacterial growth until 9 h post-inoculation, regardless of the MOIs, whereas from 10 to 12 h, the bacterial growth was moderately recovered (Figure 4A). In turn, although D10 continuously reduced the *S*. Typhimurium SJTUF 13277 count throughout the culture period, it failed to completely block the bacterial growth at most MOIs, except an MOI of 10, at which D10 abolished the growth of *S*. Typhimurium SJTUF 13277 during the first 8 h, followed by a mild rebound in bacterial growth from 9 to 12 h (Figure 4B).

### 3.5. Genomic Features of D10

**Figure 5:**
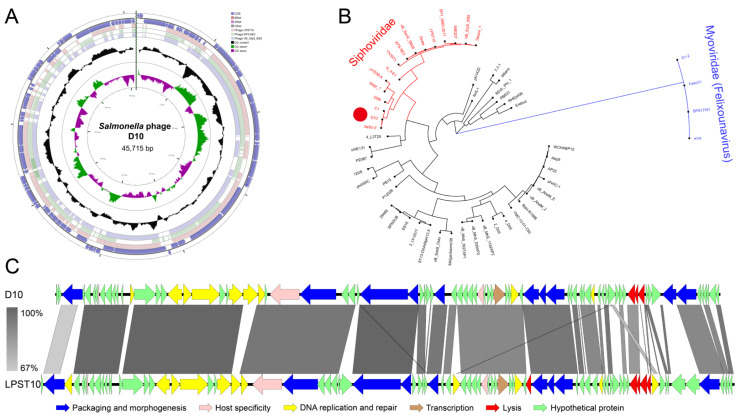
Genomic features of D10. (**A**) Circular genome map of D10 with the GC content indicated in black and the GC skew indicated in green and purple. The genome of D10 is presented by the two outermost circles, with the next three inner circles presenting genome alignment between D10 and the phages LPST10, VB_StyS_BS5, and KFS-SE2. (**B**) Phylogeny of 55 phages based on the sequences of terminase large subunit. Phage D10 is highlighted by the solid red circle. Myoviruses, including D1-2, are indicated in blue. (**C**) A sketch of the D10 genome with the denoted functional modules in comparison with LPST10. The identity between genome sequences is indicated by grey lines.

Whole-genome sequencing allowed a holistic insight into the features of D10, which presented a linear dsDNA genome with a length of 45,715 bp and a GC content of 46.1% (Figure 5A). The highest homology was evident between the genome sequence of D10 and a previously identified *Salmonella* phage, LPST10, with a coverage of 78%, followed by homology to *Salmonella* phages VB_StyS_BS5 and KFS-SE2, with coverages of 76% and 73%, respectively (Figure 5A). The large terminase subunit is generally conserved in phages and is a key factor for DNA packaging [31]. Based on this, phylogenetic study revealed that D10 is closely grouped with phages C1 and SeSz-2 belonging to the *Siphoviridae* family. This supports the morphology-based analysis (Figure 5B). The D10 genome was predicted to contain a total of 83 open reading frames (ORFs), with 26 ORFs possessing annotated functions: 10 ORFs allocated to the packaging and morphogenesis module, such as those encoding the major tail subunit and coat protein; 10 ORFs allocated to the replication and transcription module, such as those encoding DNA helicase and homing endonuclease; three ORFs involving host specificity, such as that encoding the tailspike protein; and three ORFs assigned to the lysis module, including those encoding inner membrane spanin protein Rz, class II holin, and lysozyme (Figure 5C; Table S2). No tRNAs or genes related to lysogeny, virulence, or antibiotic resistance were detected in the D10 genome (Figure 5C; Table S2).

### 3.6. Biocontrol of MDR Salmonella Enteritidis by the Phage Cocktail in Liquid Eggs

**Figure 6:**
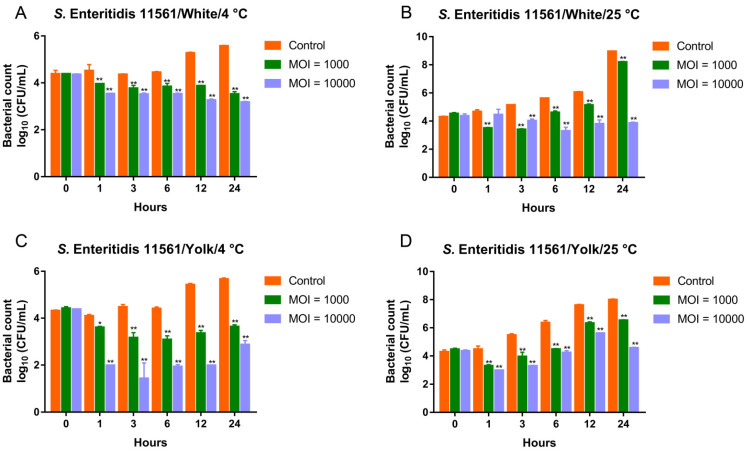
Biocontrol of MDR *S*. Enteritidis 11561 by the three-ingredient phage cocktail in liquid eggs. (**A**) Biocontrol of *S*. Enteritidis 11561 in egg whites at 4 °C. (**B**) Biocontrol of *S*. Enteritidis 11561 in egg whites at 25 °C. (**C**) Biocontrol of *S*. Enteritidis 11561 in egg yolks at 4 °C. (**D**) Biocontrol of *S*. Enteritidis 11561 in egg yolks at 25 °C. * *p* < 0.05, ** *p* < 0.01.

We previously isolated and characterized two *Salmonella* phages (D1-2 and Pu20) showing potent lytic activity toward MDR *Salmonella* strains. They belong to the *Myoviridae* and *Podoviridae* families, respectively [15,16]. Here, the newly identified siphovirus D10 was mixed with those two phages to develop a three-ingredient cocktail. The efficiency of the cocktail in controlling the prevalent MDR *S*. Enteritidis and *S*. Typhimurium strains was tested in liquid eggs (risky agents for salmonellosis [3] and matrices with high viscosity and a complicated pH [32]). For *S*. Enteritidis 11561, the cocktail significantly reduced the viable bacteria in the liquid eggs, regardless of the experimental temperature (Figure 6). In the egg whites, when added at MOIs of 1000 and 10,000, the cocktail was effective in reducing viable bacteria at both 4 and 25 °C (Figure 6A,B). Notably, when *S*. Enteritidis 11561 was treated with the cocktail in egg whites at 4 °C, a continuous reduction in bacterial counts was observed during the test period (Figure 6A). In the contaminated egg yolks, adding the cocktail at MOIs of 1000 and 10,000 successfully diminished the burden of *S*. Enteritidis 11561 during the test period at both 4 and 25 °C (Figure 6C,D).

### 3.7. Biocontrol of MDR Salmonella Typhimurium by the Phage Cocktail in Liquid Eggs

**Figure 7:**
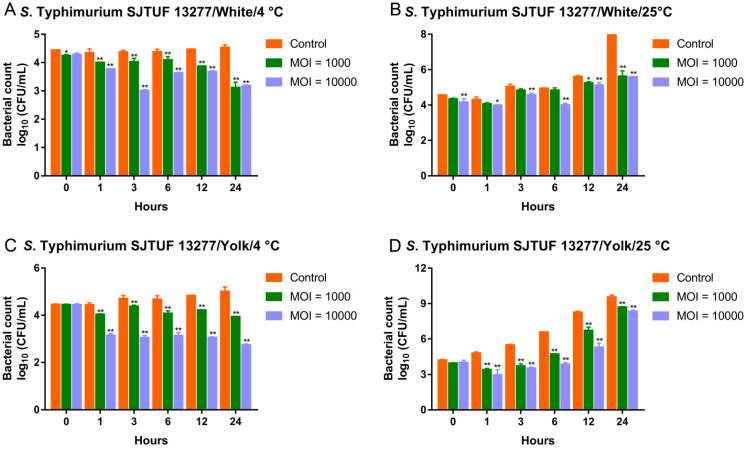
Biocontrol of MDR *S*. Typhimurium SJTUF 13277 by the three-ingredient phage cocktail in liquid eggs. (**A**) Biocontrol of *S*. Typhimurium SJTUF 13277 in egg whites at 4 °C. (**B**) Biocontrol of *S*. Typhimurium SJTUF 13277 in egg whites at 25 °C. (**C**) Biocontrol of *S*. Typhimurium SJTUF 13277 in egg yolks at 4 °C. (**D**) Biocontrol of *S*. Typhimurium SJTUF 13277 in egg yolks at 25 °C. * *p* < 0.05, ** *p* < 0.01.

The cocktail also effectively reduced viable *S*. Typhimurium SJTUF 13277 in liquid eggs (Figure 7). In the egg whites, the cocktail effectively decreased the viable bacteria at 4 °C, regardless of MOIs applied (Figure 7A). At 25 °C, the antimicrobial effects of the cocktail were measured at all designated time points at an MOI of 10,000. However, at an MOI of 1000, significant decreases in bacterial counts were only observed at 12 and 24 h (Figure 7B). In the contaminated egg yolks, the addition of the cocktail at MOIs of 1000 and 10,000 was able to reduce viable *S*. Enteritidis 11561 at all examined time points, regardless of the temperature applied (Figure 7C,D). Moreover, at 4 °C, treatment with the cocktail at an MOI of 1000 in egg whites and an MOI of 10,000 in egg yolks continuously reduced the bacterial counts during the test period (Figure 7A,C).

## 4. Discussion

Bacteriophages are envisioned as promising green approaches to controlling pathogens in the context of antimicrobial resistance [33]. In the food industry, an additional advantage of applying phages is that these natural agents have a minimal influence on organoleptic properties compared to chemical preservatives, making phages desired additives to meet the demand for naturalness [34]. The key step for the utilization of phages is to discover and characterize potential phage candidates. In this study, we characterized a *Salmonella* phage (D10) isolated from raw chicken. D10 is proposed as an effective antimicrobial tool to prevent food contamination as manifested by its high lytic activity against different MDR *Salmonella* serovars. Meanwhile, D10 showed a limited influence on bacteria from other genera, which guarantees its compatibility with constitutive microbiota in foods and humans.

Growth properties are a key parameter involving the application of phages in the biocontrol of pathogens, since a short latent period and a large burst size are considered to favor the rapid replication and effective release of phages. These two parameters can be conveyed by the one-step growth curve [35,36]. The latent period of D10 (10 min) is shorter than that of many previously identified *Salmonella* phages [16,37,38], implying its speediness in killing pathogens. D10 was shown to possess a burst size of 163 PFU/cell, which is larger than that of many other reported phages, including the two phages constituting the three-ingredient cocktail (D1-2 and Pu20) with a burst size of 104 and 34 PFU/cell, respectively [15,16,19,37]. 

Phage D10 exhibited high resistance to acidic and alkaline conditions, as manifested by its viability at a pH ranging from 4 to 12, making it a desired candidate for application in foods with a special pH, such as juice, fruits, and particularly eggs. Although the whole egg is approximately pH neutral, the egg yolk has a pH ranging from 6.0 to 6.9, whereas that of egg white is from 7.6 to 9.2 during storage [32]. In our study, all three phages used to design the cocktail were relatively stable under a range of pH conditions [15,16]. This allows them to be effective in preventing *Salmonella* contamination in matrices with distinct pH. Compared to phages D1-2 and Pu20, D10 exhibited moderate resilience to thermal stress, since it was completely inactive after incubation at 60 °C for 60 min, whereas viable phages can be detected for D1-2 and Pu20 under the same condition [15,16].

It has been proposed that the phages used for biocontrol application should be subjected to whole-genome sequencing to obtain a comprehensive insight [14]. Thus, genomic analysis of phage D10 was carried out, which showed that D10 comprises no genes involving lysogeny, pathogenicity, or antibiotic resistance, making it an attractive natural antimicrobial agent. The phylogeny and morphology indicated that D10 is a member of the *Siphoviridae* family, which was corroborated by the high homology of the D10 genome to siphovirus LPST10 [19]. Two lysis-related molecules, the spanin and holin proteins, were found to share a high similarity in D10 and LPST10. However, a considerable variation in the lysozyme sequence was observed between the two phages. In contrast, the lysozyme of D10 showed a high similarity to that of *Klebsiella* virus KpV2811, also belonging to the *Siphoviridae* family, whereas the whole genome sequence of D10 and KpV2811 exhibited very low homology with a coverage of 11%. These three lysis-related genes identified in the D10 genome may play vital roles in killing bacterial pathogens. Holin is a depolarization-inducing protein targeting the host cytoplasmic membrane, which can control the lysis timing [39]. Spanin can facilitate the disruption of cell membranes [40], and lysozyme contributes to the hydrolyzation of the peptidoglycan layer and the release of virions [41].

The three phages implicated in the designed cocktail share several similar properties, such as a high lytic activity and specificity to *Salmonella* bacteria and a lack of virulence and antibiotic resistance genes [15,16]. Moreover, they possess distinct advantages compared to one another. Phage D10 is evident for its largest burst size; D1-2 recovered from sewage showed the broadest host range [15], while Pu20 isolated from sewage exhibited the fastest adsorption rate [16], which has been proposed as an indicator for the affinity of phages to their hosts [42]. 

We tested the performance of the three-ingredient cocktail in liquid eggs, not only because eggs and their derived products are highly risky in the context of *Salmonella* infection [43], but because these foods have also been evidenced to counteract phage efficiency in killing pathogens [33]. Compared to other liquid foods such as milk and juice, liquid eggs significantly impede the antimicrobial activity of phages [44,45,46]. This might be conveyed by the high viscosity of liquid eggs, which could diminish the diffusion and homogeneous distribution of phages [44]. Another factor causing the distinct phage performance in liquid eggs might be the effect of the intrinsic properties of eggs on phage viability, since it has been revealed that the phage titer is reduced in liquid eggs during storage, whereas is increased in milk and juice under the same conditions [44]. 

Despite the aforementioned negative impact on the phage efficiency caused by liquid eggs, the three-component cocktail developed in our study significantly reduced viable *Salmonella* bacteria from two prevalent serovars, *S*. Enteritidis and *S*. Typhimurium, regardless of their drug resistance profiles. Moreover, the occurrence of phage resistance makes bacteria insensitive to phage infections and is a considerable barrier to the biocontrol application of phages [47]. When facing selective pressure, bacteria evolved multiple mechanisms to combat phages such as blocking phage adsorption, impeding phage DNA entry, and destroying phage nucleic acids [48]. Consistent with previous studies using individual phages [49,50], the emergence of resistance to phage D10 was observed in this study, as evidenced by a diminished inhibition of bacterial growth in the medium within 12 h after inoculation. It has been reported that cocktails composed of multiple phage types can mitigate phage resistance [51]. We observed that, at 4 °C, the three-ingredient cocktail could continuously reduce the bacterial burden of *S*. Enteritidis 11561 in liquid egg whites and the bacterial burden of *S*. Typhimurium SJTUF 13277 in both liquid egg whites and yolks, implying the potential of the cocktail in overcoming phage resistance. However, similar results were not evident at 25 °C. 

We noticed that the cocktail was more effective at 4 °C than at 25 °C, which is in line with previous studies demonstrating higher phage activity in reducing bacteria at 4 °C compared to relatively higher temperatures such as 20 and 25 °C [15,52,53]. One of the reasons causing such distinct phage actions at different temperatures might be that the appropriate temperature could benefit bacterial replication; in this study, a higher increase in bacterial counts in the control group was detected after incubation at 25 °C than that at 4 °C. Additionally, a relatively high temperature might directly impact phage viability, as it has been reported that the phage titer decreases more significantly in Chinese cabbage after incubation at 25 °C for 24 h compared to that at 4 °C [53].

## 5. Conclusions

In summary, in this study, we characterized a *Salmonella* phage (D10) isolated from raw chicken, which exhibited high lytic activity against MDR *Salmonella* strains from multiple serovars, but barely infected bacteria from other genera. The phage showed robust stability to different pH conditions and moderate resilience to thermal stress, as well as a short latent period and a large burst size. Genomic analysis indicated that the phage D10 is a new member of the *Siphoviridae* family and lacks genes related to lysogeny, virulence, and antibiotic resistance. Moreover, a three-ingredient phage cocktail was generated by mixing D10 with the previously identified myovirus D1-2 and podovirus Pu20. The cocktail significantly reduced the bacterial counts of MDR *Salmonella* strains in liquid eggs at different temperatures. These results demonstrate that D10 is a desired antimicrobial agent for the biocontrol of MDR *Salmonella*.

## Data Availability

The D10 genome sequence is available in the NCBI nucleotide database under an accession number MZ489634.

## Supplementary Materials

The following are available online at https://www.mdpi.com/article/10.3390/v13081626/s1, Table S1: Information on bacterial strains used in this study. Table S2: Information on hypothetical genes in the D10 genome.
