# Supplementary material for: Characterization and Application of a Lytic Phage D10 against Multidrug-Resistant Salmonella
Source: Viruses. 2021 Aug 17;13(8):1626. doi: 10.3390/v13081626 (PMC8402666; doi:10.3390/v13081626)
Supplement: Supplementary file 1 [file viruses-13-01626-s001.zip › viruses-1333995-supplementary/Table S1.pdf]

Table S1. Information on bacterial strains used in this study

Name	Description	Reference
3710	*Salmonella* Dublin, isolate	Laboratory stock
3723	*Salmonella* Dublin, isolate	Laboratory stock
10960	*Salmonella* Enteritidis, isolate, multidrug-resistant	Laboratory stock
11561	*Salmonella* Enteritidis, isolate, multidrug-resistant[1]	Laboratory stock
13500	*Salmonella* Indiana, isolate, multidrug-resistant	Laboratory stock
13520	*Salmonella* Indiana, isolate, multidrug-resistant	Laboratory stock
10855	*Salmonella* Typhimurium, isolate, multidrug-resistant	Laboratory stock
SJTUF13306	*Salmonella* Typhimurium, isolate, multidrug-resistant	Laboratory stock
SJTUF13277	*Salmonella* Typhimurium, isolate, multidrug-resistant[2]	Laboratory stock
SJTUF13336	*Salmonella* Typhimurium, isolate, multidrug-resistant	Laboratory stock
SJTUF13337	*Salmonella* Typhimurium, isolate, multidrug-resistant	Laboratory stock
SJTUF13350	*Salmonella* Typhimurium, isolate, multidrug-resistant	Laboratory stock

[1]*S.* Enteritidis 11561 was resistant to ampicillin, azithromycin, ceftriaxone, fosfomycin, nalidixic acid, streptomycin, sulfamethoxazole, and trimethoprim-sulphamethoxazole.

[2]*S.* Typhimurium SJTUF 13277 was resistant to ampicillin, chloramphenicol, ciprofloxacin, gentamicin, kanamycin, nalidixic acid, ofloxacin, streptomycin, sulfamethoxazole, trimethoprim-sulphamethoxazole, and tetracycline.
